# A phase II study of Endostatin in combination with paclitaxel, carboplatin, and radiotherapy in patients with unresectable locally advanced non-small cell lung cancer

**DOI:** 10.1186/s12885-016-2234-0

**Published:** 2016-04-11

**Authors:** Xiao-Jiang Sun, Qing-Hua Deng, Xin-Min Yu, Yong-Lin Ji, Yuan-Da Zheng, Hao Jiang, Ya-Ping Xu, Sheng-Lin Ma

**Affiliations:** Departments of Radiation Oncology, Zhejiang Cancer Hospital, 38 Guangji Road, Hangzhou, 310022 China; Departments of Radiation Oncology, Hangzhou Cancer Hospital, Hangzhou, 310002 China; Departments of Medical Oncology, Zhejiang Cancer Hospital, Hangzhou, 310022 China; Departments of Medical Oncology, Zhejiang Hospital, Hangzhou, 310013 China

**Keywords:** NSCLC, Endostatin, Concurrent chemoradiotherapy, Clinical trial

## Abstract

**Background:**

Endostatin inhibits the pro-angiogenic action of basic fibroblast growth factor and vascular endothelial growth factor in different human cancers. This study assessed the efficacy of endostatin combined with concurrent chemoradiotherapy of non-small cell lung cancer (NSCLC).

**Methods:**

Nineteen patients with unresectable stage III NSCLC, Eastern Cooperative Oncology Group (ECOG) performance status 0-l, and adequate organ function were treated with 60–66 Gy thoracic radiation therapy over 30–33 fractions concurrent with weekly 7.5 mg/m^2^ endostatin for 14 days, 50 mg/m^2^ paclitaxel, and 2 mg/mL/min carboplatin over 30 min. Patients were then treated with 7.5 mg/m^2^ endostatin for 14 days, 150 mg/m^2^ paclitaxel, and 5 mg/mL/min carboplatin every 3 weeks for 2 cycles as the consolidation treatment. The objective response rate was recorded according to the Response Evaluation Criteria in Solid Tumors (RECIST) criteria, and the toxicity was evaluated using the National Cancer Institute (NCI) Common Toxicity Criteria.

**Results:**

Six patients were unable to complete the consolidation treatment (4 pulmonary toxicity, 1 tracheoesophageal fistulae, and 1 progressive disease). Seventeen patients were included for data analysis. Specifically, one (5.9 %) patient had a complete response and 12 (70.6 %) had a partial response, whereas two patients had stable disease and the other two had disease progression. The overall response rate was 76 % (95 % confidence interval [CI], 51 %–97 %). The median progression-free survival was 10 months (95 % CI, 7.6–12.3 months), and the median overall survival was 14 months (95 % CI, 10.7–17.2 months). Early 10 patients who completed the treatment regimen showed that four patients experienced grade III pulmonary toxicity a few months after chemoradiotherapy, leading to the early closure of the trial according to the study design.

**Conclusions:**

The reslult of concurrent endostatin treatment with chemoradiotherapy in locally advanced unresectable NSCLC did not meet the goal per study design with unacceptable toxicity. The real impact of endostatin as the first-line treatment combined with chemoradiotherapy on the survival of NSCLC patients remains to be determined. (NCT 01158144).

## Background

Lung cancer is the most significant worldwide health problem, and it accounted for 1.6 million new cancer cases and 1.4 million cancer-related deaths worldwide in 2008 [1]. Histologically, lung cancer can be classified primarily as small cell lung cancer or non-small cell lung cancer (NSCLC), and up to 85 % of all lung cancer cases are NSCLC. To date, more than one-third of NSCLC cases are still diagnosed at the advanced stages of disease when curable surgery is no longer an option. A standard treatment for patients with inoperable locally advanced NSCLC is the use of concurrent chemoradiotherapy (CRT) [2, 3]. Clinically, chemoradiotherapy often fails to control NSCLC progression, and many patients die of recurrent disease. Thus, novel treatment strategies that effectively control NSCLC are urgently needed.

To this end, many research efforts have focused on developing novel treatment regimens to target tumor angiogenesis. Cancer tissues consist of a population of rapidly dividing and growing cancer cells, and to support tumor aggression, tumor cells secrete various growth factors [e.g., basic fibroblast growth factor (bFGF) and vascular endothelial growth factor (VEGF)] to induce tumor angiogenesis in order to increase the supply of oxygen and other essential nutrients within tumor tissues [4]. Endostatin, a peptide identified in 1996, can specifically inhibit the activity of bFGF and VEGF to suppress tumor-related neovascular endothelial cells and induce cancer cell apoptosis [5]. A previous clinical trial has positively evaluated endostatin application in NSCLC [6]. Two additional randomized phase II studies in China also evaluated endostatin as the first-line therapy against advanced NSCLC and showed that endostatin together with platinum-based chemotherapy might increase response rates and prolong progression-free survival (PFS) and overall survival (OS) in NSCLC patients [7, 8]. Moreover, previous data on preclinical lung cancer models demonstrated that endostatin used as an adjuvant to radiation can significantly enhance the antitumor efficacy of radiotherapy in lung cancer cells [9, 10]. Taken together, previous studies have shown in vitro and in vivo that endostatin has anti-tumor activity as both an adjuvant and a concurrent treatment for different human cancers. Thus, in this phase II clinical trial, we assessed the efficacy of combined endostatin treatment with concurrent chemoradiotherapy on patients with unresectable stage III NSCLC.

## Methods

### Patient eligibility

The study protocol was approved by the institutional review board of Zhejiang Cancer Hospital (#200934) on September 1, 2009 and registered in ClinicalTrials.gov (#NCT01158144), May 13, 2010. Patients provided written informed consent to participate in this clinical trial and were informed of the investigational nature of the trial.

In this clinical study, we prospectively recruited 19 patients with unresectable stage IIIA or IIIB NSCLC between October 2009 and December 2011. All patients with NSCLC (16 squamous and 3 adenosquamous cell lung cancer) were histologically confirmed, and all patients had Eastern Cooperative Oncology Group (ECOG) performance status 0 or l and adequate organ functions. Tumor lymph node metastasis was diagnosed by either histology, positron emission tomography with lymph node >0.5 cm in size, or computed tomography (CT) scan of 1-cm lymph nodes. The patients had no history of previous chemotherapy, radiotherapy, or surgical resection. Lung function of a forced expiratory volume in 1 s (FEV1) ≥1.5 L was also met, and the patients did not receive any full dose of anticoagulant or have any other pathologic conditions. None of the patients had a fine needle/core biopsy or mediastinoscopy within 7 days before treatment.

### Treatment of patients

**Fig. 1:**
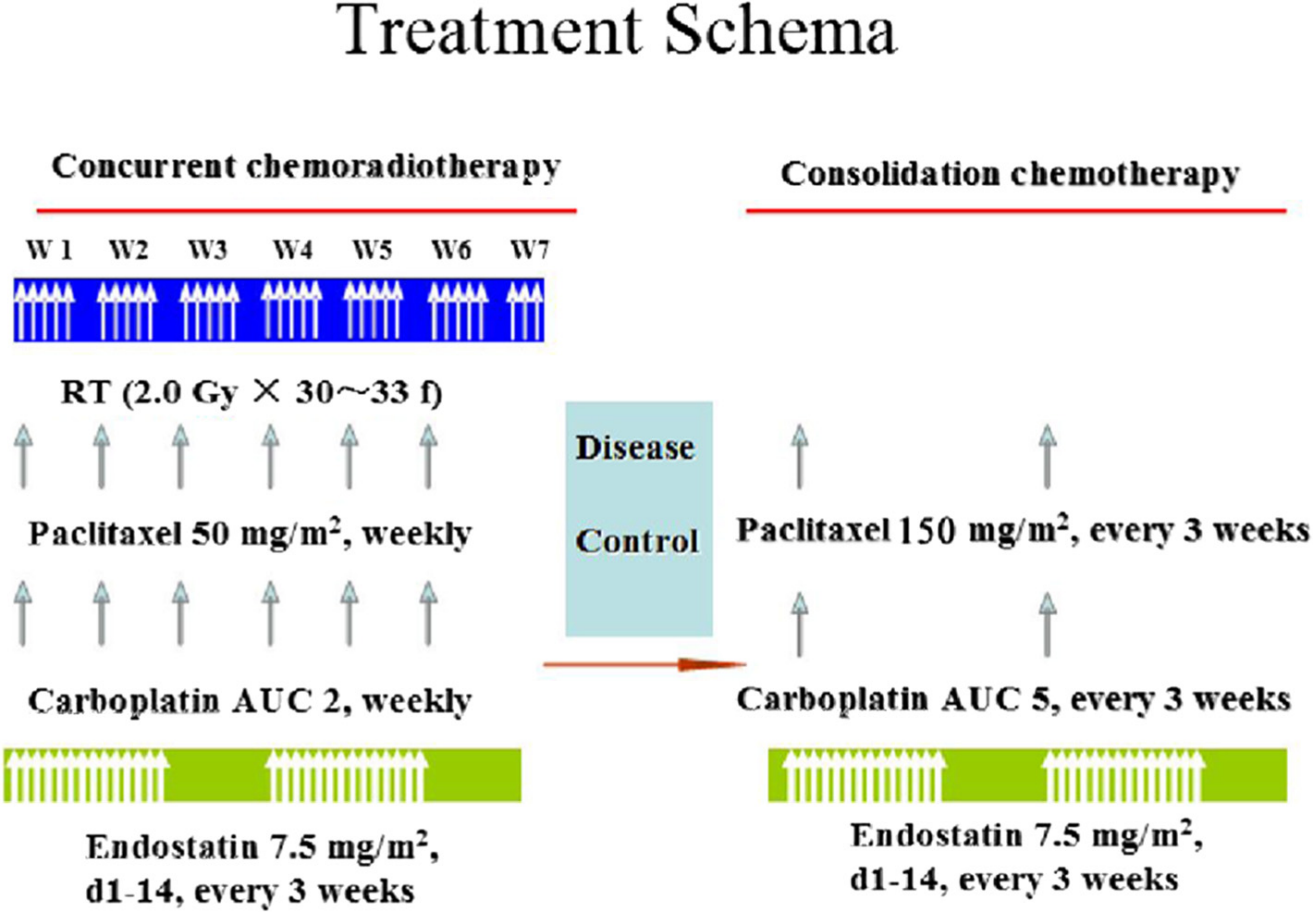
Treatment schedules of the patients

The detailed treatment plan is summarized in Fig. 1. All patients received the concurrent CRT regimen, i.e., chemotherapy consisting of weekly 50 mg/m^2^ paclitaxel over 1 h, weekly 2 mg/mL/min carboplatin over 30 min, and 7.5 mg/m^2^ endostatin over 3 h infusion between days 1 and 14 and between days 22 and 35. Radiation therapy was field arranged and determined by 3D or IMRT planning at the primary lesion and involvement of metastatic lymph nodes, and was prescribed at 60–66 Gy and given in 30–33 fractions at 200 cGy/day, 5 days a week without interruption. After 4–7 weeks of completion of radiation therapy, patients without progressive disease according to the Response Evaluation Criteria in Solid Tumors (RECIST) were then given 150 mg/m^2^ paclitaxel and 5 mg/mL/min carboplatin on day 1 and 7.5 mg/m^2^ endostatin between days 1 and 14 every 3 weeks for two cycles as the consolidation treatment.

### Toxicity evaluation and treatment modifications

In this clinical trial, we followed the National Cancer Institute (NCI) Common Toxicity Criteria version 3.0 to assess treatment-related toxicities and adverse events [http://ctep.cancer.gov/protocolDevelopment/electronic_applications/ctc.htm]. In brief, if the absolute granulocyte count was <1.5 × 10^9^/L, and/or the platelet count was <75 × 10^9^/L, chemotherapy was delayed. Dose delays up to 2 weeks from day 1 of the current cycle were permitted for recovery from adverse events. Paclitaxel and carboplatin infusion was recommenced with a 25 % dose reduction if patients experienced higher than grade II hematological toxicity during the previous treatment cycle. The dosages of paclitaxel and carboplatin were reduced to 50 % if the patients still suffered grade III hematological toxicity. A maximum of two dose reductions were permitted. Furthermore, granulocyte colony-stimulating factor at a dose of 5 μg/kg was recommenced for treating neutropenic events. Following the first dose of endostatin, toxicity was assessed weekly in all patients, and special attention was given to monitoring blood pressure, bleeding, cardiovascular events, esophagitis, and tracheoesophageal/bronchial fistulae. Modification of the endostatin dose due to drug toxicity could be made at the discretion of physicians. If one or more study drugs were discontinued, further treatment with the remaining drugs was allowed in the absence of disease progression.

### Patient evaluation and follow-up

All patients were evaluated before participation in this clinical trial according to medical history, physical exam, PS, laboratory tests, pulmonary function test, EKG, and MRI or CT scan of the brain, chest, liver, and adrenal glands. During CRT and consolidation treatment, complete blood counts were assessed weekly. Patients’ history, physical exam results, and chemistries were re-assessed prior to each treatment cycle. Once endostatin was started, weekly toxicity assessment was required and continued until 60 days after discontinuation of endostatin or until all adverse events had resolved. Treatment response was assessed at the end of CRT and carboplatin/paclitaxel/endostatin consolidation treatment and then every 2–3 months for 2 years and every 6 months until 5 years.

### Statistical analyses

This is a prospective phase II study at a single institution, and the primary end point of the trial was to evaluate the response rate and toxicity of this concurrent radiotherapy and chemotherapy regimen. The secondary end point was PFS and OS of the patients with unresectable locally advanced NSCLC. This clinical trial was designed to measure a response rate [complete responses (CRs) and partial responses (PRs)] of 85 % compared to a minimal, clinically meaningful response rate of 70 %. Using an alpha = 0.05 and a power of 80 %, the target number of patients required to achieve an 85 % CR plus PR rate was 20 patients according to a previous study [11].

Given the known risks of concurrent chemoradiotherapy, a toxicity analysis was planned after 10 patients had completed all treatment regimes. Toxicity was assessed as unacceptable if 4 or more of these 10 patients experienced at least grade III esophageal or pulmonary toxicity.

PFS was calculated from the day of initiation of treatment to the date of disease progression, death  or until the last follow-up, whereas OS was calculated from the first day of CRT until death or until the last follow-up. The survival curves were calculated using the Kaplan–Meier method. The considered variables included: age, gender, histopathology, smoking status (cigarettes/year), and stage. All statistical analyses were performed using SPSS software, Version 19.0 (SPSS Inc., Chicago, IL).

## Results

### Patient characteristics

This study was opened in October 2009 and closed in December 2011 according to the protocol definition, i.e., early 10 patients who completed treatment, including four or more patients with at least grade III pulmonary toxicity a few months after the CRT, thus leading to the early closure of the trial according to the study design..

The characteristics of these 19 assessable patients are shown in Table 1. In brief, there were 16 patients with squamous cell carcinoma and a higher proportion of male patients with stage IIIB. Seventeen patients completed the CRT, whereas six patients were unable to complete the consolidation treatment (4 due to pulmonary toxicity, 1 due to tracheoesophageal fistulae, and 1 due to progressive disease).

**Table 1:** Characteristics of patients

Characteristics		No. of patients (*N* = 19)
Age (years)	Median	58
	Range	36–65
	<60 years	10
	≥60 years	9
Gender	Male	16
	Female	3
Cigarettes/year	≥400	15
	Never smoked	4
Histology	SCC	16
	ADC	3
UICC stage	IIIA	5
	IIIB	14

### Treatment efficacy

Nineteen patients had measurable disease at baseline. Response was not assessable in two (10.5 %) patients due to pulmonary toxicity and incompletion of the CRT as planned. The objective tumor response for targeted lesions was assessed and calculated 2–4 weeks after the concurrent therapy. Seventeen patients were included in data analysis. The overall response rate (CR + PR) was 76 % (95 % CI: 51 %–97 %), two (12 %) patients had stable disease, and two (12 %) patients had disease progression (Table 2).

**Table 2:** Treatment efficacy in patients (*N* = 17)

Treatment efficacy	No. of patients	%
Response		
Overall response rate	13	76.4
Disease control rate	15	88.2
Complete response	1	5.9
Partial response	12	70.6
Stable disease	2	11.7
Progressive disease	2	11.7
Survival		
Median PFS (months)	10.0 (95 % CI: 7.6–12.3 months)	
Median OS (months)	14.0 (95 % CI: 10.7–17.2 months)	

**Fig. 2:**
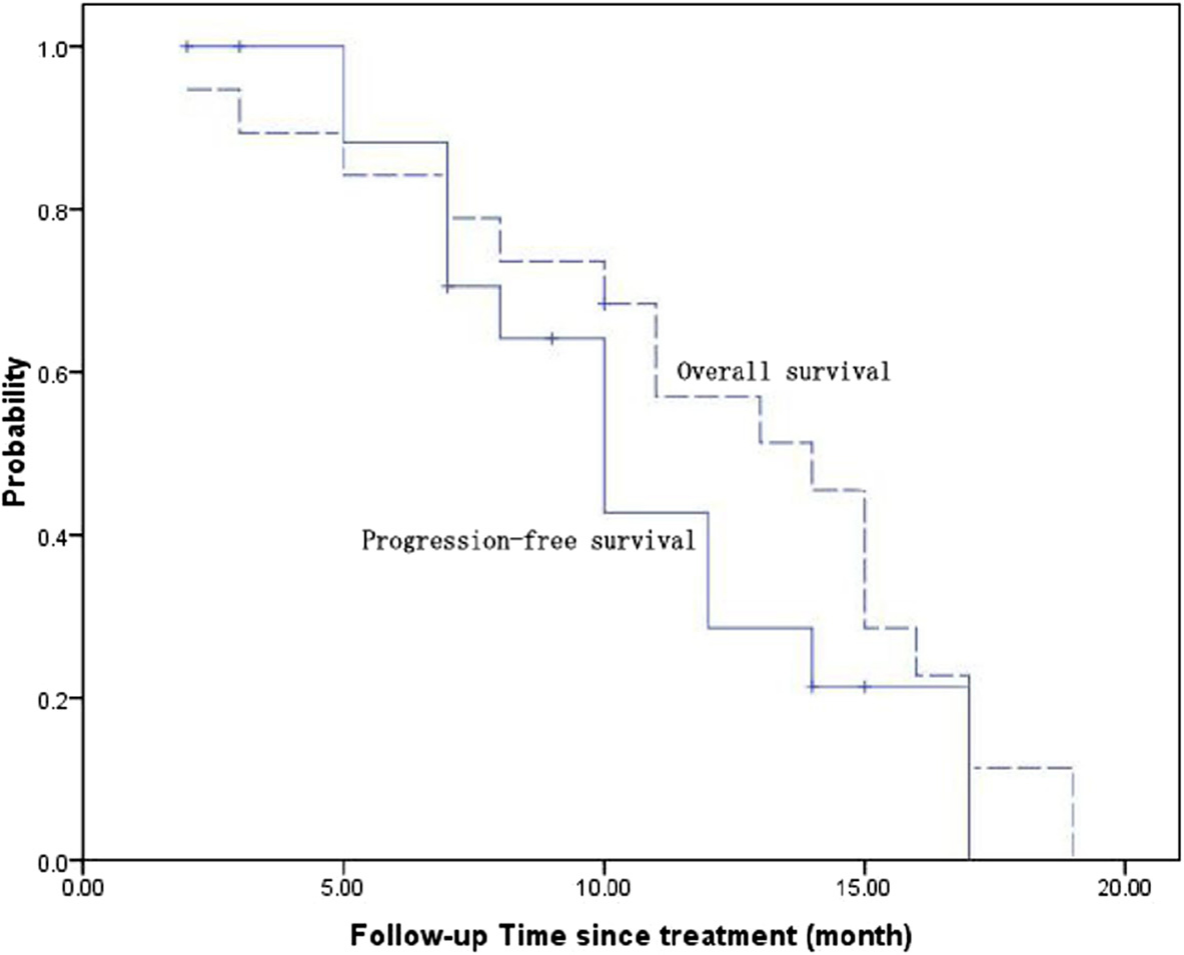
Kaplan–Meier survival curves of progression-free survival and overall survival of patients. The median progression-free survival was 10 months (95 % CI, 7.6–12.3 months), and the median overall survival was 14 months (95 % CI, 10.7–17.2 months)

With a median follow-up time of 36 months, the median PFS was 10.0 months (95 % confidence interval [CI]: 7.6–12.3 months), and the median OS was 14.0 months (95 % CI: 10.7–17.2 months; Fig. 2).

### Treatment toxicity

Toxicities did occur during and after the concurrent CRT or the consolidation treatment (Table 3). Specifically, grades III or IV leukopenia or neutropenia was the most common toxicity, occurring in 21 % and 26 % of the patients, respectively. Moreover, 11 % patients developed Grade III/IV febrile neutropenia, 16 % patients had grade III or IV esophagitis, and 26 % (5 episodes) had grade III pneumonitis. In addition, there was one patient with treatment-related tracheoesophageal/bronchial fistulae (one patient developed grade III esophagitis during concurrent CRT and then developed bracheoesophageal/bronchial fistulae 19 days after the first cycle of consolidation treatment).

**Table 3:** Toxicity profile after treatment

Complications	0	I	II	III	IV	III + IV, % (n)
	%(n)	% (n)	% (n)	% (n)	% (n)	
Hematological toxicities						
Anemia	32 (6)	26 (5)	32 (6)	11 (2)	0	11 (2)
Leukopenia	21 (4)	32 (6)	26 (5)	16 (3)	5 (1)	21 (4)
Neutropenia	16 (3)	32 (6)	26 (5)	21 (4)	5 (1)	26 (5)
Thrombocytopenia	26 (5)	42 (8)	21 (4)	5 (1)	5 (1)	11 (2)
Non-hematological toxicities						
Nausea	37 (7)	37 (7)	16 (3)	11 (2)	0	11 (2)
Vomiting	47 (9)	32 (6)	11 (2)	11 (2)	0	11 (2)
Anorexia	53 (10)	26 (5)	11 (2)	5 (1)	5 (1)	11 (2)
Hemorrhage	90 (17)	5 (1)	5 (1)	0	0	0
Fatigue	37 (7)	32 (6)	26 (5)	5 (1)	0	5 (1)
ALT/AST	89 (15)	16 (3)	5 (1)	0	0	0
Arrhythmia	63 (12)	37 (7)	0	0	0	0
Esophagitis	26 (5)	37 (7)	16 (4)	11 (2)	5 (1)	16 (3)
Pneumonitis	21 (4)	26 (5)	26 (5)	26 (5)	0	26 (5)

## Discussion

Chemotherapy has been successfully incorporated with radiation to treat unresectable locally advanced NSCLC with an acceptable toxicity. However, the treatment outcome remains largely unsatisfactory, indicating that novel agents to additively or synergistically enhance the action of radiation against NSCLC are urgently needed. Thus, our current study determined the efficacy of combined endostatin treatment with concurrent chemoradiotherapy in patients with unresectable stage III NSCLC. We found that out of the 17 patients, 1 had a complete response and 12 had a partial response. Two patients had stable disease, and another two had disease progression. The median PFS was 10 months, and the median OS was 14 months. Early 10 patients who completed the treatment regimen showed that four patients experienced grade III pulmonary toxicity, leading to the early closure of the trial according to the study design. Thus, our current data showed that concurrent endostatin treatment with chemoradiotherapy in locally advanced unresectable NSCLC did not meet the goal per study design with unacceptable toxicity. The real impact of endostatin as the first-line treatment combined with chemoradiotherapy on the survival of NSCLC patients remains to be determined.

Currently, platinum-based CRT represents the standard treatment regime for locally advanced NSCLC patients, although the treatment efficacy is constrained by poor local control and radiation-induced lung injury (RILI). To improve the effect of the platinum-based CRT on NSCLC, a number of clinical trials have been conducted, but similar to our current study, the results suggest that the concomitant treatment of patients with unresectable locally advanced NSCLC with endostatin, paclitaxel/carboplatin, and radiotherapy does not show a significant clinical value. The present study indicated that the overall response rate (76 %; 95 % CI: 51 %–97 %) did not exceed the goal per study design (85.0 %), and the toxicity analysis after 10 patients had completed treatment indicated that four patients had grade III pulmonary toxicity. With a median follow-up time of 36 months, the median PFS was 10 months, and the median OS was 14 months. Similar results were reported by another phase II study which assessed the activity and safety of weekly paclitaxel/carboplatin vs. cisplatin/etoposide based CRT for patients with unresectable IIIA/IIIB NSCLC. The overall response rates (CR + PR) were 81.3 % in the paclitaxel/carboplatin arm. With a median follow-up time of 46 months, the median OS was 13.5 months (95 % CI, 8.3–18.7 months) in the paclitaxel/carboplatin group [12].

Furthermore, endostatin is a 20-kDa COOH-terminal proteolytic fragment derived from the basement membrane component of collagen XVIII. Endostatin is one of the most potent inhibitors of angiogenesis. It was originally identified from the supernatant of a murine hemangioendothelioma cell line as an inhibitor of proliferation of endothelial cell proliferation and of angiogenesis [13–15]. Indeed, several in vitro and in vivo studies demonstrated that endostatin may potentiate radiation [9, 10]. Thus, endostatin was designed as a rational therapeutic agent combined with CRT for treatment of unresectable locally advanced NSCLC. However, endostatin has toxicity concerns and may cause development of tracheoesophageal/bronchial fistulae in NSCLC patients, but this is generally an uncommon event resulting from CRT of lung cancer. In a simultaneous and ongoing study in limited stage SCLC, among 29 patients there were 2 confirmed and 1 suspected episode of tracheoesophageal/bronchial fistulae [16, 17]. All 3 patients had grade III esophagitis during CRT and bevacizumab treatment (another tumor angiogenesis inhibitor that is a humanized monoclonal antibody directed against VEGF). Subsequently, an additional patient developed a fatal tracheoesophageal/bronchial fistulae during maintenance treatment. In an independent study of NSCLC treatment, 2 of 5 patients developed tracheoesophageal/bronchial fistulae during maintenance treatment with chemotherapy and bevacizumab [17]. Both patients also had severe esophageal toxicity after CRT and bevacizumab. Therefore, together these results imply that severe esophageal toxicity as a result of this treatment may predispose patients to the development of tracheoesophageal/bronchial fistulae. In the Socinski trial, the rate of grade III/IV esophagitis was 29 %, and one case of tracheoesophageal/bronchial fistulae developed 3 months after CRT [18]. One patient in our study developed grade III esophagitis during concurrent CRT and then developed bracheoesophageal/bronchial fistulae 19 days after the first cycle of consolidation treatment. Thereafter, our study was amended to exclude patients with grade III or higher esophagitis from receiving endostatin. There were no cases of bracheoesophageal/bronchial fistulae in two previously randomized phase II studies in China in which endostatin was given with platinum-based chemotherapy as first-line treatment [7, 8].

However, our current data showed that it was difficult to ascertain whether addition of endostatin was efficacious in treating these patients and whether there was a synergy of endostatin plus radiation therapy. The median PFS and OS estimated at 10 and 14 months, respectively, were similar to those of previous clinical trials of CRT alone [1, 2, 4–6, 10, 16]. Whether the inconsistent schedule of endostatin from other studies made our work unsuccessful is worthy of more research. Our current study is also limited by a small study population, and a future study with more patients could help us to clarify the effects of endostatin on patients with unresectable locally advanced NSCLC.

## Conclusion

We were unable to successfully integrate endostatin into concurrent CRT for unresectable locally advanced NSCLC because of safety, and the data are insufficient to determine efficacy. There are numerous new molecularly targeted agents that are of interest in the treatment of NSCLC. Careful study design and close toxicity monitoring is imperative to properly integrate their use in multimodality therapy.

## Abbreviations

bFGF: basic fibroblast growth factor
CRs: complete responses
CRT: chemoradiotherapy
CT: computed tomography
ECOG: Eastern cooperative oncology group
FEV1: forced expiratory volume in 1 s
NSCLC: non-small cell lung cancer
OS: overall survival
PFS: progression-free survival
PRs: partial responses
RECIST: response evaluation criteria in solid tumors
RILI: radiation-induced lung injury
VEGF: vascular endothelial growth factor
